# Incidence and Outcomes of Acute Respiratory Distress Syndrome: A Nationwide Registry-Based Study in Taiwan, 1997 to 2011

**DOI:** 10.1097/MD.0000000000001849

**Published:** 2015-10-30

**Authors:** Wei Chen, Yih-Yuan Chen, Ching-Fang Tsai, Solomon Chih-Cheng Chen, Ming-Shian Lin, Lorraine B. Ware, Chuan-Mu Chen

**Affiliations:** From the Department of Life Sciences, National Chung Hsing University, Taichung (WC, CMC); Division of Pulmonary and Critical Care Medicine, Chia-Yi Christian Hospital, Chiayi (WC, MSL); College of Nursing, Dayeh University, Changhua (WC); Department of Respiratory Therapy, China Medical University, Taichung (WC); Department of Internal Medicine, Chia-Yi Christian Hospital (YYC); Department of Medical Research, Ditmanson Medical Foundation Chia-Yi Christian Hospital (CFT, SCCC); Department of Respiratory Care, Chang Gung University of Science and Technology, Chiayi Campus, Chiayi, Taiwan (MSL); Departments of Medicine and Pathology, Microbiology and Immunology, Vanderbilt University School of Medicine, Nashville, TN (LBW); and Rong-Hsing Translational Medicine Center, and iEGG Center, National Chung Hsing University, Taichung, Taiwan (CMC).

## Abstract

Most epidemiological studies of acute respiratory distress syndrome (ARDS) have been conducted in western countries, and studies in Asia are limited. The aim of our study was to evaluate the incidence, in-hospital mortality, and 1-year mortality of ARDS in Taiwan.

We conducted a nationwide inpatient cohort study based on the Taiwan National Health Insurance Research Database between 1997 and 2011. A total of 40,876 ARDS patients (68% male; mean age 66 years) were identified by International Classification of Diseases, 9th edition coding and further analyzed for clinical characteristics, medical costs, and mortality.

The overall crude incidence of ARDS was 15.74 per 100,000 person-years, and increased from 2.53 to 19.26 per 100,000 person-years during the study period. The age-adjusted incidence of ARDS was 15.19 per 100,000 person-years. The overall in-hospital mortality was 57.8%. In-hospital mortality decreased from 59.7% in 1997 to 47.5% in 2011 (*P* < 0.001). The in-hospital mortality rate was lowest (33.5%) in the youngest patients (age 18–29 years) and highest (68.2%) in the oldest patients (>80 years, *P* < 0.001). The overall 1-year mortality rate was 72.1%, and decreased from 75.8% to 54.7% during the study period. Patients who died during hospitalization were older (69 ± 17 versus 62 ± 19, *P* < 0.001) and predominantly male (69.8% versus 65.3%, *P* < 0.001). In addition, patients who died during hospitalization had significantly higher medical costs (6421 versus 5825 US Dollars, *P* < 0.001) and shorter lengths of stay (13 versus 19 days, *P* < 0.001) than patients who survived.

We provide the first large-scale epidemiological analysis of ARDS incidence and outcomes in Asia. Although the overall incidence was lower than has been reported in a prospective US study, this may reflect underdiagnosis by International Classification of Diseases, 9th edition code and identification of only patients with more severe ARDS in this analysis. Overall, there has been a decreasing trend in in-hospital and 1-year mortality rates in recent years, likely because of the implementation of lung-protective ventilation.

## INTRODUCTION

Acute respiratory distress syndrome (ARDS) is a syndrome of acute respiratory failure that is characterized by alveolar–capillary barrier leakage, lung edema formation, pulmonary epithelial cell death, and an acute inflammatory response that manifests with poor lung compliance, hypoxemia, and bilateral infiltrates on chest radiograph.^1,2^ A variety of clinical disorders are associated with the development of ARDS, including pneumonia, aspiration of gastric contents, sepsis, trauma, and the transfusion of blood products.^3^ Acute respiratory distress syndrome is a major clinical problem that contributes to the death of more than 70,000 people annually in the United States.^3,4^ In addition, patients who survive ARDS have reduced exercise capacity and health-related quality of life and have increased costs and use of health care services during the 5 years after discharge from the intensive care unit.^4–8^

Although this syndrome has a considerable impact on public health, relevant large-scale epidemiologic investigations have been rare in recent years. Before 1990, when a uniform definition of ARDS was not in place, several studies showed that the incidence of ARDS was approximately 1.5 to 8.3 per 100,000 person-years.^9–12^ After the American–European Consensus Conference published a uniform definition for ARDS in 1994,^2^ several studies conducted in the United States, Australia, and Europe showed that the incidence of ARDS was as high as 13.5 to 28 per 100,000 person-years.^13–17^ Subsequently, Rubenfeld et al^4^ conducted a prospective population-based cohort study in 21 hospitals in and around King County, Washington, and found that the incidence of ARDS was 78.9 per 100,000 person-years in the United States and that the incidence was age dependent and increased from 16 per 100,000 person-years for those 15 to 19 years of age to 306 per 100,000 person-years for those 75 to 84 years of age.^4^ Epidemiological studies conducted in Asia, however, are limited.^18^

Because race may be a risk factor for development of ARDS ^19^ and there has been no large-scale study of ARDS conducted in a predominantly Asian population, we conducted a retrospective cohort study using nationwide population-based data from the National Health Insurance Research Database (NHIRD) of Taiwan. The aim of our study was to investigate the incidence, medical costs, in-hospital mortality, and 1-year mortality of ARDS during a 14-year period.

## MATERIALS AND METHODS

We conducted a population-based study using data obtained from all admission records of the NHIRD. In Taiwan, the National Health Insurance program, implemented in 1995, provides compulsory health insurance that covers more than 99% of the population. National Health Insurance Research Database includes almost all outpatient and inpatient medical records, including information on patient characteristics, such as age, sex, dates of clinical visits, date of admission, and diagnostic codes. The cases of ARDS were obtained from whole-population inpatient data in NHIRD between 1997 and 2011 and were defined using International Classification of Diseases, 9th Revision, Clinical Modification (ICD-9-CM) codes. This study has been reviewed and approved by the Institutional Review Board of the Ditmanson Medical Foundation Chia-Yi Christian Hospital, Taiwan.

### Study Subjects and Definition

Patients who were hospitalized with a diagnosis of ARDS (ICD-9-CM codes 518.82, 518.5) for the first time between 1997 and 2011 were enrolled in the study. Patients whose sex was not identified or who were less than 18 years old were excluded from this study because ARDS in children has different epidemiology and outcomes.^20,21^ Demographic characteristics, resource utilization, clinical features, in-hospital mortality, and 1-year mortality were studied. Because participation in the national health insurance system in Taiwan is mandatory, patients who withdrew from the system for at least 6 months were regarded as dead. The date of withdrawing is regarded as the date of death. Age at the time of the first diagnosis was categorized into 7 groups: 18 to 29, 30 to 39, 40 to 49, 50 to 59, 60 to 69, 70 to 79, and over 80 years old. Comorbidities that were recorded included pneumonia (ICD-9-CM codes 480–488), sepsis (ICD-9-CM codes 0.38, 785.52, 790.7, 995.91, 995.92, and 995.93), trauma (ICD-9-CM code 518.5), and acute pancreatitis (ICD-9-CM code 577.0).

### Statistical Analysis

The incidence rates (per 100,000 person-years) of ARDS were calculated from 1997 to 2011 and were plotted for each age group and calendar year for both sexes. The number of ARDS patients was used as the numerator of the incidence rate, and the total population of Taiwan was used as the denominator. The total population for each year was obtained from the Department of Statistics in the Ministry of the Interior of Executive Yuan in Taiwan. The age-adjusted incidence rate per 100,000 person-years was age-adjusted to the World population in 2000. We tested for temporal trends in ARDS incidence by Poisson regression analysis. Differences in demographic characteristics, clinical features, and resource utilization of patients by survival status were tested with Student *t* test or the Wilcoxon rank sum test for continuous variables and the χ^2^ test for categorical variables. We also reported in-hospital mortality and 1-year mortality across each calendar year, and the temporal trend was tested by the Cochran–Armitage trend test. Data analysis was performed with SPSS software, Version 21 of the SPSS System for Windows (version 21.0: IBM Corporation, Somers, NY). A 2-tailed *P* value less than 0.05 was considered statistically significant.

## RESULTS

A total of 40,876 newly diagnosed ARDS patients (67.9% male; mean age 66 years) were enrolled in the study. Among them, 57.8% (n = 23,612) died during hospitalization. Patients who died during hospitalization were older (69 ± 17 versus 62 ± 19 years, *P* < 0.001) and more likely to be male (69.8% versus 65.3%, *P* < 0.001). In addition, patients who died during hospitalization had significantly higher medical costs (6421 versus 5825 US Dollars, *P* < 0.001) and shorter lengths of stay (13 versus 19 days, *P* < 0.001) than patients who survived. The most common etiologic comorbidities for ARDS were pneumonia (49.7%), followed by sepsis (33.2%), and trauma (29.9%). Some patients had more than one etiologic comorbidity. There were significant differences in etiology by hospital mortality status (Table 1).

**TABLE 1:**
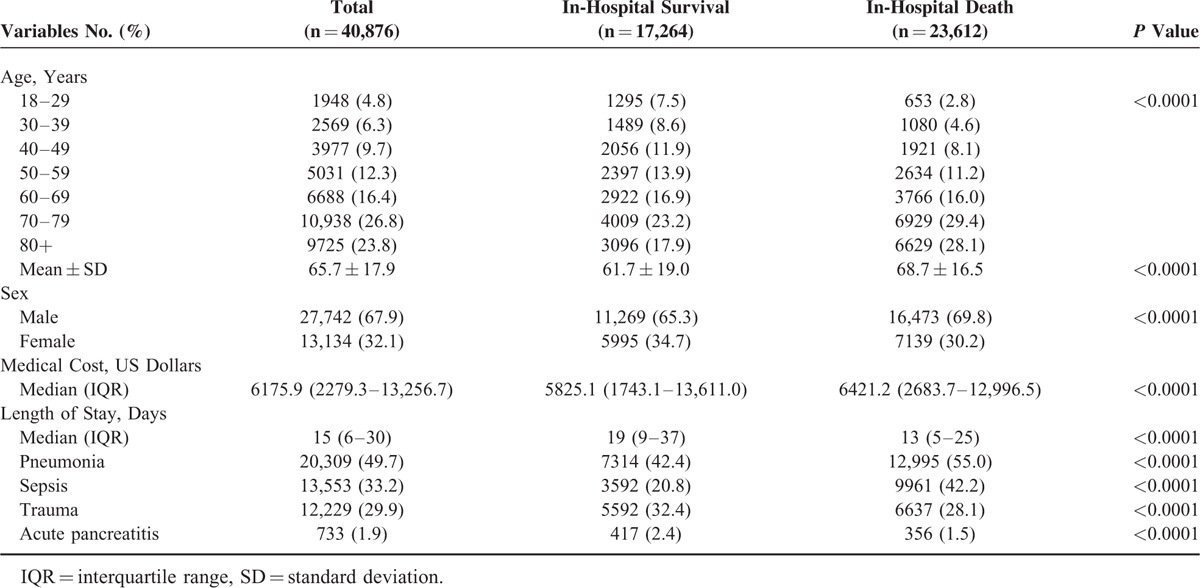
Demographic Characteristics, Clinical Features, and Resource Utilizations of Patients With Adult Respiratory Distress Syndrome

### The Trend of Incidence of Acute Respiratory Distress Syndrome

**FIGURE 1:**
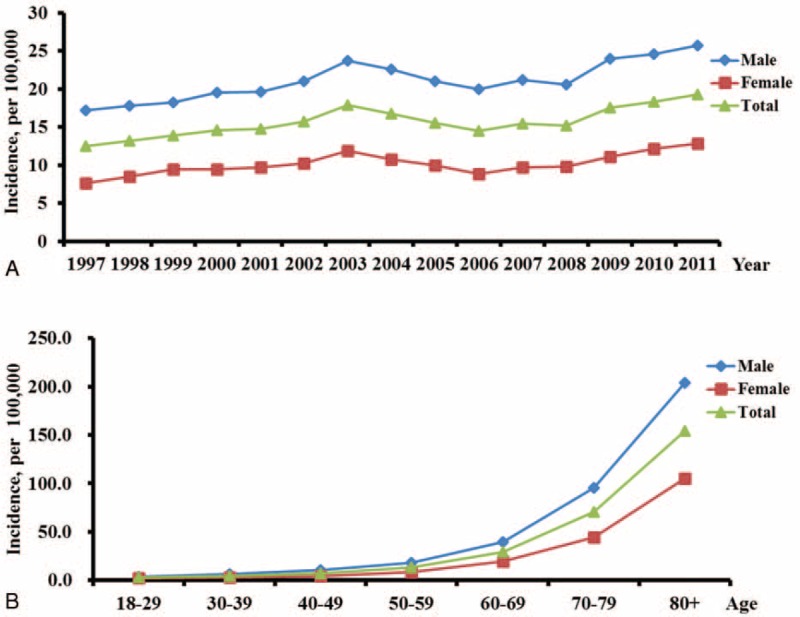
**Incidence of ARDS in Taiwan**. (A) The incidence trends of ARDS over the study period 1997–2011; (B) Age-specific incidence rates of ARDS. Blue diamonds: male; red squares: female; green triangles: total population.

**FIGURE 2:**
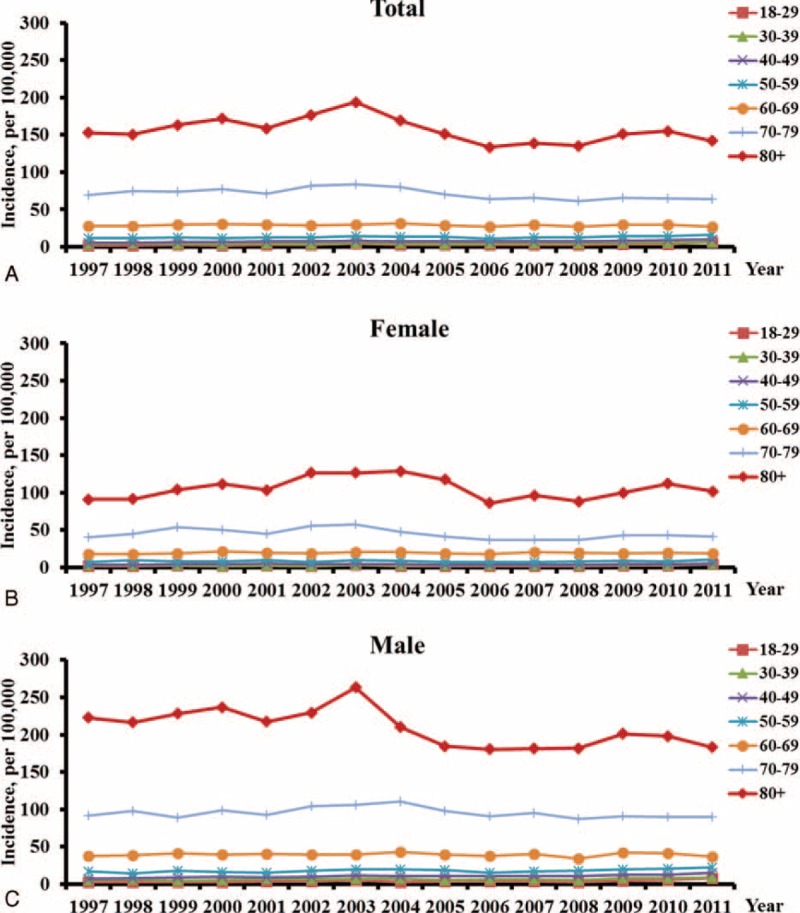
**Age- and Gender-specific incidence of ARDS in Taiwan, 1997–2011**. (A) Total population; (B) Male; (C) Female.

The estimated incidence of ARDS from 1997 to 2011 is shown in Figure 1A. The incidence of ARDS during the study period for total male and female populations was 15.74, 21.97, and 10.20 per 100,000 person-years, respectively. The age-adjusted incidence of ARDS was 15.19 per 100,000 person-years. The incidence increased from 12.53 to 19.26 per 100,000 person-years in the total population (*P* < 0.0001 by trend test). It also increased from 17.21 to 25.73 per 100,000 person-years in the male population and from 7.64 to 12.87 per 100,000 person-years in the female population (Fig. 1A). Figure 1B shows the age- and sex-specific incidence rates for ARDS in Taiwan. Overall, the incidence rate increased from 13.27 per 100,000 person-years in the group of 50 to 59 years of age to an estimated 154.05 per 100,000 person-years in the group of 80 years of age and above. The age-specific incidence rates increased with advancing age, with a sharp increase occurring in patients over the age of 70 that was evident in both men and women. As shown in Figure 2, men had higher incidence of ARDS than women in all age groups.

### In-Hospital Mortality

**FIGURE 3:**
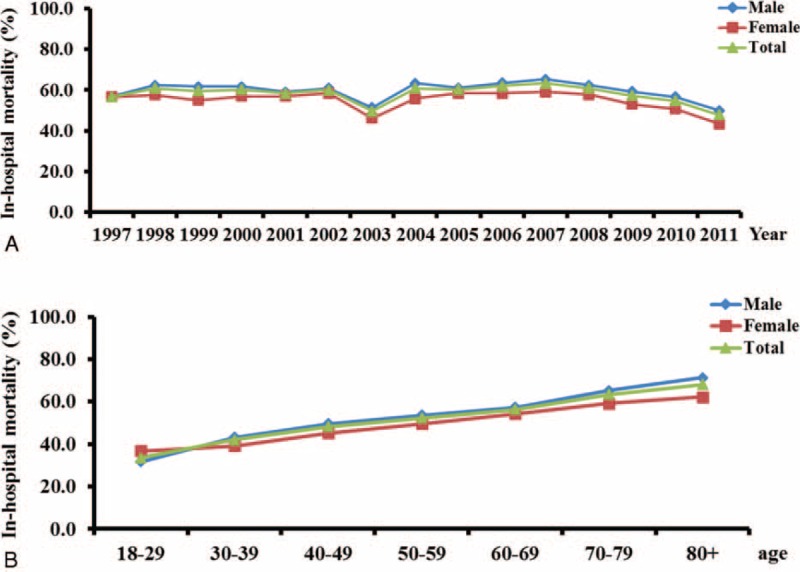
**In-hospital mortality rates of ARDS in Taiwan**. (A) In-hospital mortality rates of ARDS over the study period 1997–2011; (B) Age-specific in-hospital mortality rates of ARDS. Blue diamonds: male; red squares: female; green triangles: total population.

The overall in-hospital mortality rate of ARDS patients during the study period was 57.8%. Figure 3A shows a significant trend of a decreasing in-hospital mortality rate, from 59.7% in 1997 to 47.5% in 2011 (trend test, *P* < 0.001) in the overall population, and similar trends in both sexes. An abrupt decrease in the in-hospital mortality rate in 2003 was coincident with an outbreak of severe acute respiratory syndrome that year.^22^Figure 3B shows a significant increase in in-hospital mortality rate from 33.52% in the group of 18 to 29 years of age to 68.16% in the group of 80 years of age and above (trend test, *P* < 0.001).

### One-Year Mortality

**FIGURE 4:**
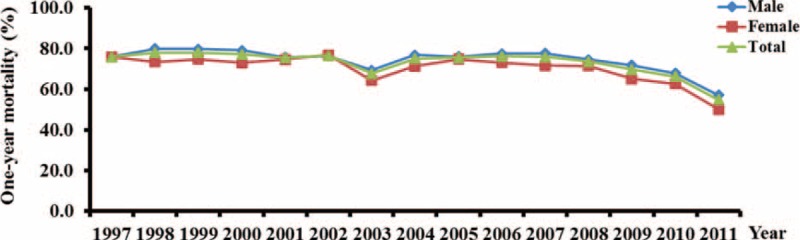
**One-year mortality rates of ARDS between 1997 and 2011 in Taiwan**. Blue diamonds: male; red squares: female; green triangles: total population.

The pattern of 1-year mortality of ARDS was very similar to that of in-hospital mortality in this study (Fig. 4**)**. The overall 1-year mortality rate of ARDS was 72.1%. We observed a trend of decreasing 1-year mortality rates from 75.8% in 1997 to 54.7% in 2011 in patients with ARDS (trend test, *P* < 0.001). There were no sex differences in 1-year mortality.

## DISCUSSION

To our knowledge, this is the first large epidemiological study of ARDS in a predominantly Asian patient population. The average incidence of ARDS was 15.74 per 100,000 person-years, and increased from 12.53 to 19.26 per 100,000 person-years during the study period. The average in-hospital mortality rate was 57.8% and decreased significantly from 59.7% to 47.5% during the study period. The in-hospital mortality rate was lowest (33.5%) in the youngest patients (age 18–29 years) and highest (68.2%) in the oldest patients (age > 80 years, *P* < 0.001). The overall 1-year mortality rate was 72.1%, and decreased from 75.8% to 54.7% during the study period.

This study used International Classification of Diseases, 9th edition (ICD-9) coding to identify patients admitted with a new diagnosis of ARDS. Several previous studies have used the ICD-9 coding system to investigate the incidence and outcomes of ARDS. Reynolds et al^23^ reported that the estimated incidence of ARDS in Maryland was in the range of 10 to 14 cases per 100,000 people and the mortality rate was 36% to 52%, using ICD-9 codes 518.5 and 518.82.^23^ Other studies have also used the ICD-9 coding system to determine the incidence or prevalence of ARDS in particular populations, such as those with traumatic brain injury,^24^ spinal cord injury,^25^ and subarachnoid hemorrhage.^26^ Because the ICD-9 coding system is linked to the Taiwanese healthcare reimbursement system, the accuracy of ICD-9 coding is quite precise. Indeed, more than 1000 studies using the Taiwan NHIRD have been published, including a number of important epidemiologic observations.^27–31^ In the case of ARDS, the specificity of ICD-9 coding is likely improved by the fact that the diagnosis is typically made only by intensivists or pulmonologists. We, however, acknowledge that reliance on ICD-9 coding to identify patients with ARDS likely underestimates the true incidence of ARDS, especially with regard to mild or moderate ARDS. This may explain why the observed incidence of ARDS in this study is lower than that reported by Rubenfeld et al^4^ in a study that used prospective ARDS phenotyping by trained investigators.

Overall, the in-hospital mortality in this cohort was considerably higher than mortality reported in other large cohorts.^1,4^ There are several potential explanations. One possibility is that use of ICD-9 coding identifies a more severely ill group of patients than other methods of ARDS phenotyping. The in-hospital mortality rate in 2011 (47%) in the current study is very close to the mortality reported for patients with severe ARDS in the report of the Berlin Definition of ARDS, (45%),^4^ which accounted for approximately one-third of the total study participants in the Berlin study.^32,33^ One possible interpretation is that the Taiwanese study population is representative of severe ARDS and that ICD-9 coding missed many of the less severe mild and moderate cases. If this were the case, then the calculated incidence of all ARDS in Taiwan might be closer to 60 per 100,000 person-years, which is close to the recent report in the United States.^4^ In support of this estimate, Moss et al used multiple-cause mortality data to analyze the incidence of ARDS in a population that was more likely to be in the severe stage of the disease. That study showed that the incidence of ARDS was 17 to 26 per 100,000 person-years,^34^ which is very close to our finding. Another potential explanation for the high mortality in the Taiwanese cohort is that factors related to health care delivery or patient race lead to the differences in observed outcomes.

As shown in Figure 2, men had higher incidence of ARDS than women in all age group. One potential explanation is that the rate of alveolar fluid clearance is faster in women with acute lung injury compared with men, which might lead to more rapid resolution of pulmonary edema.^35^ In addition, cigarette smoking has recently been shown to be a risk factor for ARDS ^36,37^ and men are 11-fold more likely to smoke than women in Taiwan.^38^ Unfortunately, the NHIRD does not contain information about patient smoking.

Although a number of experimental studies have shown promising benefits in treating ARDS, no clinical studies have demonstrated an effective pharmacologic treatment.^39^ The reported mortality of ARDS ranges from 30% to 75% depending on the patient population.^40^ Several studies have shown a decrease in ARDS mortality over time,^41–43^ mainly because of the implementation of new ventilator strategies.^41^ A protective lung strategy could reduce the risk of further lung injury, systemic inflammation, and subsequent multisystem organ failure in ARDS patients.^42,44–46^ Interestingly, in the current study, there was an abrupt decrease in mortality in 2003, which coincided with the outbreak of severe acute respiratory syndrome in Asia ^22^ and an increase in incidence of ARDS (Fig. 1A). A possible explanation may be that physicians in Taiwan that year were more aware of ARDS and provided better care and were more adherent to low tidal volume ventilation in ARDS patients.

This study has both strengths and limitations. The major strengths include the large number of ARDS patients in the NHIRD as well as the long period of follow-up. To our knowledge, this is the first nationwide epidemiological study of ARDS, and the follow-up period is the longest available, so we can clearly see the trend of the disease. The limitations of the study are inherent to ICD-09 database without any specified definition of ARDS and retrospective in nature. We did not have detailed data for definition of ARDS, such as the chest radiograph reports, ratio of arterial oxygen partial pressure to fractional inspired oxygen, and utility of positive end-expiratory pressure. In addition, major indexes as Acute Physiology and Chronic Health Evaluation score, Sequential Organ Failure Assessment score, or Lung Injury Score were lacking. Second, because we were limited to the ICD-9 coding data for each admission, we could not identify the actual etiologies of ARDS in this study. Finally, no clinical data such as arterial blood gas analyses were available to grade the severity of ARDS.

In conclusion, we provide the first study of large-scale epidemiological data for ARDS in Asia. The incidence of ARDS may be underestimated because of the use of ICD-9 coding in the NHIRD and severe ARDS may be overrepresented. Nevertheless, the study provides valuable new information on the incidence and outcomes of ARDS in an Asian patient population. Consistent with findings in other countries, there has been a decrease in in-hospital and 1-year mortality rates in recent years that likely reflects the benefits of lung protective mechanical ventilation.
